# LSEC Fenestrae Are Preserved Despite Pro-inflammatory Phenotype of Liver Sinusoidal Endothelial Cells in Mice on High Fat Diet

**DOI:** 10.3389/fphys.2019.00006

**Published:** 2019-02-12

**Authors:** Edyta Kus, Patrycja Kaczara, Izabela Czyzynska-Cichon, Karolina Szafranska, Bartlomiej Zapotoczny, Agnieszka Kij, Agnieszka Sowinska, Jerzy Kotlinowski, Lukasz Mateuszuk, Elzbieta Czarnowska, Marek Szymonski, Stefan Chlopicki

**Affiliations:** ^1^Jagiellonian University, Jagiellonian Centre for Experimental Therapeutics, Kraków, Poland; ^2^Jagiellonian University, Faculty of Physics, Astronomy, and Applied Computer Science, Centre for Nanometer-Scale Science and Advanced Materials, Kraków, Poland; ^3^Jagiellonian University Medical College, Chair and Department of Toxicology, Kraków, Poland; ^4^The Children’s Memorial Health Institute, Warsaw, Poland; ^5^Jagiellonian University, Faculty of Biochemistry, Biophysics and Biotechnology, Department of General Biochemistry, Kraków, Poland; ^6^Jagiellonian University Medical College, Chair of Pharmacology, Kraków, Poland

**Keywords:** LSEC metabolism, LSEC inflammation, LSEC porosity, non-alcoholic fatty liver disease, liver steatosis

## Abstract

Healthy liver sinusoidal endothelial cells (LSECs) maintain liver homeostasis, while LSEC dysfunction was suggested to coincide with defenestration. Here, we have revisited the relationship between LSEC pro-inflammatory response, defenestration, and impairment of LSEC bioenergetics in non-alcoholic fatty liver disease (NAFLD) in mice. We characterized inflammatory response, morphology as well as bioenergetics of LSECs in early and late phases of high fat diet (HFD)-induced NAFLD. LSEC phenotype was evaluated at early (2–8 week) and late (15–20 week) stages of NAFLD progression induced by HFD in male C57Bl/6 mice. NAFLD progression was monitored by insulin resistance, liver steatosis and obesity. LSEC phenotype was determined in isolated, primary LSECs by immunocytochemistry, mRNA gene expression (qRT-PCR), secreted prostanoids (LC/MS/MS) and bioenergetics (Seahorse FX Analyzer). LSEC morphology was examined using SEM and AFM techniques. Early phase of NAFLD, characterized by significant liver steatosis and prominent insulin resistance, was related with LSEC pro-inflammatory phenotype as evidenced by elevated ICAM-1, E-selectin and PECAM-1 expression. Transiently impaired mitochondrial phosphorylation in LSECs was compensated by increased glycolysis. Late stage of NAFLD was featured by prominent activation of pro-inflammatory LSEC phenotype (ICAM-1, E-selectin, PECAM-1 expression, increased COX-2, IL-6, and NOX-2 mRNA expression), activation of pro-inflammatory prostaglandins release (PGE_2_ and PGF_2α_) and preserved LSEC bioenergetics. Neither in the early nor in the late phase of NAFLD, were LSEC fenestrae compromised. In the early and late phases of NAFLD, despite metabolic and pro-inflammatory burden linked to HFD, LSEC fenestrae and bioenergetics are functionally preserved. These results suggest prominent adaptive capacity of LSECs that might mitigate NAFLD progression.

## Introduction

The LSECs, belonging to the group of liver non-parenchymal cells, comprise approximately 20% of the total number of liver cells (Sørensen et al., 2015). LSECs are characterized by a unique morphology and specialized function which differentiate them from other endothelial cells in the body (Aird, 2007). Moreover, the unique LSEC ability to eliminate a variety of macromolecules from the blood circulation by receptor-mediated endocytosis makes LSECs highly specialized scavenger cells (Sørensen et al., 2012). Although the scavenger activity of LSECs has been widely investigated, the potential significance of their barrier function is still neglected and knowledge about the role of LSEC defenestration in liver diseases is still limited.

In healthy liver, the presence of LSEC fenestrae and lack of basement membrane do not represent a barrier for macromolecule transport in the same sense as vascular endothelial cells in other organs. Still, because of the active regulation of the passage of a wide range of solutes and macromolecules between blood and hepatocytes, LSECs present a ‘dynamic, functional barrier’ protecting the liver parenchyma (Fraser et al., 1995; Sørensen et al., 2015). Several studies have stressed the importance of LSEC defenestration as the main characteristic or even early event in the development of liver pathology (Poisson et al., 2017). The lack of LSEC fenestrae directly contributes to intrahepatic resistance and hepatocellular damage (Francque et al., 2012). Furthermore, maintaining LSECs’ fenestrae-dependent blood filtration is thought to be particularly important for the hepatic metabolism of lipids (Fraser et al., 1995). Defenestration has been shown to impair the hepatic uptake of various lipoproteins that may lead to severe hyperlipoproteinemia and liver steatosis (Clark et al., 1988; Herrnberger et al., 2014). Le Couteur et al. (2002) have suggested that there is a link between the defenestration commonly associated with aging and impaired clearance of cholesterol rich chylomicron remnants in elderly people, increasing the risk for development of lipid abnormalities and atherosclerosis. Accordingly, LSEC defenestration may represent an important pathogenic mechanism contributing to NAFLD development. This disease, ranging from simple steatosis, through steatosis with inflammation and fibrosis (NASH) may result in cirrhosis or hepatocellular carcinoma. Moreover, NAFLD (affecting more than 25% of the global population) is closely related with obesity (51%), hyperlipidemia (69%), insulin resistance (22.5%), and hypertension (39%) (Younossi et al., 2016).

A number of studies have suggested the involvement of LSEC dysfunction in NAFLD (Maslak et al., 2015a,b). Functional studies showed that the impairment in NO-dependent vasodilation response to insulin in the isolated liver was present as soon as after 3 days of HFD in rats, implying that LSEC dysfunction may be one of the earliest features of NAFLD (Pasarín et al., 2011). Moreover, NAFLD-related impairment of LSEC function was linked to increased hepatic portal perfusion pressure and downregulation of the Akt/eNOS pathway that preceded liver inflammation and fibrosis (Pasarín et al., 2012). On the other hand, liver steatosis may induce adaptive, anti-inflammatory LSEC response, as evidenced by a decrease in pro-inflammatory chemokine production in LSECs as opposed to hepatocytes producing more pro-inflammatory chemokines in response to free fatty acids (McMahan et al., 2016).

Liver sinusoidal endothelial cell fenestrae formation and their regulation is a dynamic process that is closely associated with the LSEC cytoskeleton (Wisse, 1972; Steffan et al., 1987; Braet et al., 1995; Braet et al., 1996b; Zapotoczny et al., 2018). However, the exact mechanism responsible for LSEC defenestration remains to be elucidated (Venkatraman and Tucker-Kellogg, 2013; Herrnberger et al., 2014; Hunt et al., 2018). Interestingly, reports suggest that regulation of fenestrae size directly depends on LSEC bioenergetic status (i.e., ATP depletion leads to LSEC defenestration) (Braet et al., 2003; Zapotoczny et al., 2017c).

Given the fact that it is not entirely clear whether LSEC dysfunction always coincides with defenestration, we revisited the relationship between LSEC pro-inflammatory response, defenestration and impairment of LSEC bioenergetics in NAFLD in mice. For that purpose, we simultaneously characterized inflammatory response, morphology as well as bioenergetics of LSECs in early and late phases of HFD-induced NAFLD.

## Materials and Methods

### Animals and Experimental Design

In the experiments, 6-week old male C57Bl/6 mice were fed control (AIN-93G) or HFD (60 kcal% of fat) (Research Diets, United States) for 2, 4, and 8 weeks (*n* = 3–4/group/each experimental time-point) reflecting the early stage of NAFLD and 15 and 20 weeks (*n* = 6–8/group/each experimental time-point) reflecting the late phase of the disease. Mice dedicated for assessment of *in vivo* NAFLD progression and LSEC bioenergetics were obtained respectively from animal research facilities of the Nofer Institute of Occupational Medicine in Łodz (Poland) for early stage of NAFLD and from the Medical University of Bialystok (Poland) for the late phase of NAFLD. In turn, mice for assessment of LSEC structure and molecular biology research were purchased from animal research facilities of the Medical University of Bialystok (Poland). The number of mice dedicated for LSEC isolation was at least three animals per group. Animals were housed in colony cages in a temperature-controlled environment (22–25°C) with a 12 h light/dark cycle. Mice had free access to food and water. At the end of the experiment, the mice were weighted to obtain final body mass and anesthetized with ketamine (100 mg/kg) and xylazine (10 mg/kg) administered intraperitoneally.

All procedures involving animals were conducted according to the Guidelines for Animal Care and Treatment of the European Union and were approved by the I Local Animal Ethics Commission at Jagiellonian University in Kraków, Poland (Permit No. 292/2015).

### Blood Biochemistry

At the end of each experimental time-point, blood was collected under fasting conditions (4 h) from the left ventricle of the mice heart and placed into plastic tubes containing 20 I.U./ml heparin. Plasma was obtained by blood centrifugation (1,000 × *g* for 10 min) and used for the following measurements: CHOL, HDL, LDL, TGs, ALT, and AST. These parameters were measured by the enzymatic photometric method using an automatic biochemical analyzer Pentra 400 (Horiba, Japan) according to the manufacturer’s instructions.

### Histological Evaluation of Liver Steatosis

Fragments of liver tissue were fixed in 4% buffered formalin. One fragment was prepared according to the standard paraffin method and stained with hematoxylin and eosin (HE) for general histology and immune cell infiltration and PSR for collagen deposition (Kus et al., 2018), while the second fragment was immersed in a 30% sucrose solution overnight for cryoprotection and afterward frozen in Tissue-Tek^®^OCT (optimum cutting temperature) medium at −80°C. Frozen sections were cut into 7-μm thick sections, stained with ORO for fat deposition (Kus et al., 2018) and photographed under × 100 magnification. At least six images of each section were randomly obtained. The images were subsequently analyzed in terms of steatosis by using the Columbus Image Data Storage and Analysis System (Perkin Elmer, United States) with an algorithm adapted for ORO stained sections.

### Assessment of Insulin Resistance

Fasting plasma glucose concentration was measured by the enzymatic photometric method using an automatic biochemical analyzer Pentra 400 (Horiba, Japan) according to the manufacturer’s instructions. A GTT was performed at the end of each experimental time-point in fasting (4 h) mice injected intraperitoneally with glucose solution (2 g/kg of body weight). Blood was collected from the tail veins before (0 min) and at 15, 30, 45, 60, and 120 min after glucose administration. Blood glucose concentrations were measured using a glucometer (Accu-Check, Roche Diagnostic, France). For assessing the GTT, the AUC of blood glucose concentration was calculated geometrically by applying the trapezoidal method (Venn and Green, 2007).

### Protocol of LSECs Isolation

The primary LSECs were isolated from C57Bl/6 mice fed for 2–20 weeks HFD according to a protocol presented by Smedsrød and Pertoft (1985) with modification (Kochan et al., 2017; Zapotoczny et al., 2018). Briefly, liver was initially perfused with perfusion buffer (37°C) in order to purify it from the blood and then with buffered collagenase (Liberase TM (Roche)). Parenchymal cells (hepatocytes) were removed using low-speed centrifugation at 50 × *g*. The LSECs were isolated by gradient density sedimentation of resting non-parenchymal cells on 25–50% Percoll and purified using LSEC-specific CD146-based isolation on magnetic MicroBeads (MACS, MiltenyiBiotec, Germany). The LSEC fraction yielded approximately 2–8 × 10^6^ LSECs per liver with a purity of >95% cells, as determined by SEM and AFM microscopy and high acetylated LDL endocytosis.

Isolated LSECs were incubated overnight in EBM-2 Basal Medium (Lonza) supplemented with 1% Antibiotic Antimycotic Solution for Cell Culture (Sigma-Aldrich), under 37°C and 5% CO_2_ conditions to spread out, unless otherwise stated. Isolated LSEC were used for experiments within 24 h from the isolation.

### LSEC Immunocytochemistry

Isolated LSECs after overnight incubation were washed with PBS, fixed with 4% formaline solution (10 min), slightly permeabilized using 0.1% Triton-X (5 min), then preincubated for 30 min with blocking buffer containing 5% normal goat serum (Jackson Immuno) and 2% dry milk. Immunofluorescent staining was performed for 1 h using rat anti-E selectin Ig (Invitrogen), rabbit anti-CD31 Ig, rat anti-ICAM1 Ig (Abcam) or mouse anti-eNOS Ig (Sigma-Aldrich), followed by goat-anti-rabbit, goat-anti-rat or goat-anti-mouse Cy3-conjugated secondary antibodies (Jackson Immuno) applied for 30 min. Cells stained with mouse antibodies were previously incubated with MOM blocking reagent (Vector) to reduce unspecific binding. Hoechst 33258 (Sigma-Aldrich) was used for nuclei counterstaining. Serial cell images were taken using an Olympus ScanR automated fluorescence microscope (Olympus), stored as integrated datasheets and analyzed automatically by Columbus software (Perkin Elmer).

### qRT-PCR of Isolated LSECs

Total RNA was isolated from LSECs after overnight culture at 37°C in an atmosphere containing 5% O_2_ using a modified guanidinium isothiocyanate method (Chomczynski and Sacchi, 2006). Briefly, cells were lysed in fenozol (A&A Biotechnology), and after addition of chloroform, the aqueous phase containing RNA was collected into new eppendorf tubes. Next, RNA was precipitated, centrifuged, washed in ethanol and finally dissolved in nuclease-free water. RNA concentration was measured with an ND-1000 spectrophotometer (Thermo Fisher Scientific). Reverse transcription was performed using 0.5 μg of total RNA with random hexamers and M-MLV reverse transcriptase (Promega) according to the vendor’s instructions. Gene expression was measured by real-time PCR (Eco, Illumina) with a SybrGreen master mix (A&A Biotechnology) according to the protocol: 95°C for 5 min followed by 40 cycles of melting at 95°C – 20 s, annealing at 58–62°C – 20 s, elongation at 72°C – 30 s. The relative quantification of gene expression was calculated with the 2^−ΔΔCt^ method. Primer sequences, annealing temperatures and length of PCR products are listed in Table 1.

**Table 1:** Sequences of primers used in qRT-PCR analysis of gene expression.

Gene	NCBI accession no.	Forward 5′–3′	Reverse 5′–3′	T_A_	Product (bp)
*Ptgs1*	NM_008969.4	ATTGCACATCCATCCACTCCC	AGTTGTCGAGGCCAAAGCG	62	196
*Ptgs2*	NM_011198.4	CTTTGCCCAGCACTTCACCC	GGGGATACACCTCTCCACCAA	64	191
*Cybb*	NM_007807.5	AAGTTCGCTGGAAACCCTCC	GCCAAAACCGAACCAACCTC	62	88
*Il6*	NM_031168.2	ACTTCACAAGTCGGAGGCTT	GGTACTCCAGAAGACCAGAGG	64	220
*Zc3h12a*	NM_153159.2	CAGCCTCGACCAGATGTGCC	CAGCCGCTCCTCGATGAAGC	62	237
*Ef2*	NM_001961	GACATCACCAAGGGTGTGCAG	TTCAGCACACTGGCATAGAGGC	62	214

*T_A_, annealing temperature.*

### Quantification of Production of Prostaglandins by LSECs

Prostaglandin production was assessed in EBM-2 (Lonza) medium from LSECs after overnight incubation at 37°C in an atmosphere containing 5% O_2_ according method partially described by Kij et al. (2016). In details, samples were spiked with deuterated internal standards mixture (PGE_2_-d_4_, PGD_2_-d_4_, PGF_2α_-d_4_, 6-keto-PGF_1α_-d_4_) and purified via liquid-liquid extraction technique using ethyl acetate acidified with 0.13% AA (v/v). After shaking, samples were centrifuged, and the organic layer was collected and evaporated to dryness under nitrogen stream at 37°C. The dry residues were reconstituted in EtOH and samples were injected into the UPLC-MS system. The quantification of selected prostaglandins (PGE_2_, PGD_2_, PGF_2α_, 6-keto-PGF_1α_) was performed using a UFLC Nexera liquid chromatograph system (Shimadzu, Kyoto, Japan) coupled to a QTrap 5500 triple quadrupole mass spectrometer (Sciex, Framingham, MA, United States). Samples were injected onto an Acquity UPLC BEH C18 (3.0 mm × 100 mm, 1.7 μm, Waters, Milford, MA, United States) analytical column and analytes were separated under gradient elution applying 0.1% formic acid in acetonitrile (A) and 0.1% formic acid in water (v/v) (B) as mobile phases. The detection of eluted prostaglandins was carried out using negative ion electrospray ionization mass spectrometry in the multiple reaction monitoring mode (MRM) for all analytes and their deuterated internal standards. The ion transitions selected for quantification of studied prostaglandins and used internal standards were as follows: PGE_2_ (351.1→315.1), PGD_2_ (351.1→315.1), PGF_2α_ (353.1→193.0), 6-keto-PGF_1α_ (369.2→163.0) and PGE_2_-d_4_ (355.5→275.2), PGD_2_-d_4_ (355.5→275.2), PGF_2α_-d_4_ (357.4→197.3), 6-keto-PGF_1α_-d_4_ (373.3→167.1).

Data acquisition was performed under optimized conditions: spray voltage: −4500 V, source temperature: 500°C, curtain gas: 25 psi, ion source gas 1: 40 psi, ion source gas 2: 50 psi.

### Assessment of Thromboxane B_2_ and Nitrite Production Using Isolated Perfused Liver Set Up

A monovascular perfusion technique of liver perfusion was used in this study as described previously (Kus et al., 2017). The portal perfusion was performed in a recirculation manner with Krebs–Hanseleit buffer (118.0 mM NaCl, 2.52 mM CaCl_2_, 1.16 mM MgSO_4_, 24.88 mM NaHCO_3_, 1.18 mM KH_2_PO_4_, 4.7 mM KCl, 10.0 M glucose, 2.0 mM pyruvic acid, and 0.5 mM EDTA) at a constant flow rate of 2.7 ml/min/g of liver. Buffer was continuously heated to 37°C and oxygenated with 95% O_2_ and 5% CO_2_. To evaluate organ viability, activity of liver enzymes (ALT, AST, LDH) was measured in the output effluent using the Pentra 400 automatic biochemical analyzer (Horiba, Kyoto, Japan), according to the manufacturer’s instructions.

The concentration of nitrite was measured in liver perfusate after a stabilization period. Nitrite concentration was analyzed using a NOx analyzing system, ENO-20 (Eicom, Kyoto, Japan) based on a liquid chromatography method with post-column derivatization with Griess reagent as it was described by Kus et al. (2015).

The thromboxane B_2_ (TXB_2_) concentration was measured in liver perfusate obtained at 0 min (after stabilization period) and after 30 min of perfusion of isolated liver. The quantitative determination of TXB_2_ (a stable derivative of TXA_2_) in those samples was performed with a commercially available ELISA kit (Enzo Life Sciences, United States) according to manufacturer’s instruction. Data are presented as 30 min liver TXB_2_ release (ΔTXB_2_) calculated from differences between TXB_2_ concentrations at time 30 min and 0 min (Δ*TXB*_2_ = *t*_30 min_ *conc.* – *t*_0 min_*conc.*).

### Assessment of LSEC Fenestrae Using SEM

Isolated LSECs plated on coverslips were incubated overnight in EBM-2 medium (Lonza) at 37°C in an atmosphere containing 5% CO_2_. Afterward, LSECs were fixed in 2% glutaraldehyde/0.1 M sodium cacodylate/0.1 M sucrose overnight and dehydrated in ethanol gradients of 35 and 70% in 0.1 M sucrose, followed by 95 and 100% ethanol (15 min each). Dried samples were coated with gold using spatter Coater (Jeol JFC-1200 Fine Coater). Cells were imaged with a SCM (Jeol JSM – 7600) at a voltage of 5.0 kV. Microphotograph analysis revealed that among isolated LSECs, the number of cells with shrunk cytoplasm was increased in the HFD group; therefore, for further morphometric analysis, only LSECs with preserved morphology were concerned. The LSEC fenestra measurements were performed from SEM microphotographs taken under a magnification of x5,000 (to characterize the cell morphology and determine the cell area without nuclei), x7,500 (to estimate the number fenestrae and their total area in the cell) and x15,000 (to define consecutive areas in which the diameter of each fenestra was then measured). All measurements were made using the CellSense morphometric program (Olympus).

### Assessment of LSEC Fenestrae Using AFM Imaging

Freshly isolated LSECs were seeded onto uncoated glass coverslips in EGM-2 medium (Lonza) and incubated overnight at 37°C in an atmosphere containing 5% CO_2_. Afterward, LSECs were fixed with 1% glutaraldehyde for 2 min to determine the porosity and fenestra diameter distribution. All measurements were performed within 1 week. The AFM imaging was conducted in phosphate-buffered saline with Mg^2+^ and Ca^2+^ (PBS) using Nanowizard 3 JPK Instruments in a commercial liquid cell with the temperature controller (25°C).

Porosity and fenestrae diameter distributions were determined according to a procedure described previously (Zapotoczny et al., 2017a,b,c). Briefly, for imaging we used a novel fast AFM imaging mode, so-called Quantitative Imaging (QI, JPK Instruments), in which force versus distance curves are acquired point-by-point over a selected scanning area in times of milliseconds each and translated into a topographical image. The loading forces were adjusted to a particular image and probe and were within the range of 200–600 pN. Collected data were processed with JPK Data Processing Software and Fiji ImageJ software (NIH, Bethesda, MD, United States).

Fenestra diameter distribution was determined by performing high-magnification images of single sieve plates. Scanning areas were in the range of 10s of square micrometers, but point-to-point imaging resolution was always set below 15 nm. Cantilevers with sharpened tips (2–12 nm) were used for such measurements. We measured the diameter of fenestrae which were open (i.e., we could always reach the glass slide in the interior of fenestrae). Images from different LSECs isolated from at least two mice (control and HFD-fed mice), were used to create histograms of fenestra size distribution.

Porosity was calculated using large QI AFM images of a few cells selected from LSECs organized on a coverslip in a monolayer or in groups of five or more. Image sizes and point-to-point resolutions were as follows: 40 × 40 to 50 × 50 μm and 512 × 512 to 800 × 800 lines. Because of limited point-to-point resolution of such scans (62–78 nm per pixel), we were not able to properly estimate fenestrae size and area directly from the large images. Therefore, in order to calculate the porosity of LSECs, at first, we counted the number of fenestrae in each large image manually, using a Cell Counter under Fiji software, and then using the fenestrae size distribution and assuming that fenestrae are circular, we calculated the area which is occupied by the fenestrae (for details see Zapotoczny et al., 2017b). We excluded areas of cell nuclei and gaps larger than 400 nm in diameter. While doing so, we ensured that calculation of porosity is not affected by the location of the selected area. Consequently, we determined the porosity as a ratio of the area occupied by the fenestrae to the total area where fenestrae could be formed. Every point on the graph from Figure 6, therefore, represents a single AFM image, which illustrated 2–3 cells.

### Assessment of LSEC Bioenergetics

Metabolic analyses were performed using an Agilent Seahorse XFe96 Analyzer (Seahorse Bioscience, North Billerica), which enables real-time, simultaneous measurement of OCR (reflecting mitochondrial respiration) and ECAR (reflecting glycolysis) as described by Kaczara et al. (2015) with some modifications. Briefly, LSECs were seeded into Seahorse XFe96 plates 18 h prior to the experiment at a density of 70,000 cells per well, which was selected as optimal based upon a preliminary experiment and incubated overnight under 37°C and 5% CO_2_ conditions. On the day of the experiment, the cells were washed twice and incubated for 1 h prior to the beginning of the assay at 37°C in the absence of CO_2_ within bicarbonate-free low buffered XF assay media supplemented with 1 g/l of glucose, 2 mM glutamine, and 1 mM pyruvate (pH = 7.4) for the Agilent Seahorse XF Mito Stress Test or with 2 mM glutamine (glucose-free, pH = 7.4) for the Agilent Seahorse XF Glycolysis Stress Test. The media were prepared freshly on the day of the experiment. In the XF Cell Mito Stress Test, mitochondrial respiration was assessed overtime, followed by addition of oligomycin (1 μg/ml), FCCP (2 μM) and a mixture of rotenone (1 μM) and antimycin A (1 μM), respectively (concentrations of mitochondrial modulators were optimized based upon a preliminary experiment), allowing for determination of basal respiration, ATP turnover, proton leak and maximal respiratory capacity. In the XF Glycolysis Stress Test, glycolysis was assessed overtime followed by addition of glucose (10 mM), oligomycin (1 μg/ml) and 2DG (50 mM) respectively, allowing for determination of basal glycolysis and maximal glycolytic capacity.

### Statistical Analysis

Results are presented as mean ± SD. The assessment of normality and homogeneity of variances was performed using the Shapiro–Wilk and Levene’s tests, respectively. Based on the results, the non-parametric Mann–Whitney *U*-test or parametric Student’s *t*-test was used to assess the statistical significance of ^∗^*P* < 0.05, ^∗∗^*P* < 0.01, and ^∗∗∗^*P* < 0.001 between time-matched experimental groups. The results were analyzed using STATISTICA 12.0.

## Results

### NAFLD Development in HFD-Fed Mice

**FIGURE 1:**
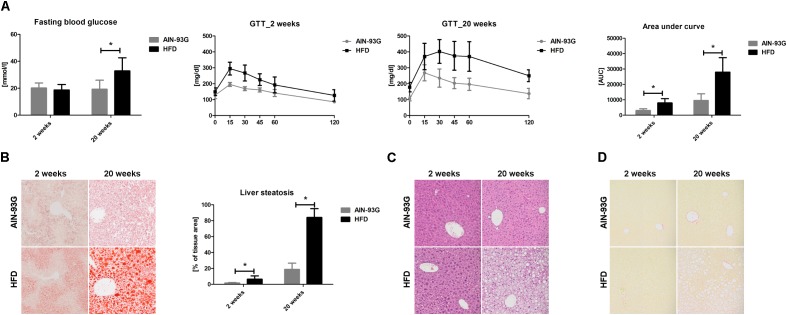
Fasting glucose concentration and GTT with calculated AUC of the GTT **(A)**. Representative images of liver steatosis (ORO staining) **(B)**, immune cells infiltration (HE staining) **(C)** and fibrosis (Picro Sirius Red staining) **(D)** and summary results of liver steatosis **(B)**, performed in C57Bl/6 mice at early and late stage of NAFLD progression. Values are means ± SD. ^∗^*P* < 0.05, ^∗∗^*P* < 0.01, ^∗∗∗^*P* < 0.001 significantly different from HFD feeding time-matched AIN-93G group values. AIN-93G, AIN-93G diet fed group; HFD, high fat diet fed group.

Early and late phases of NAFLD at 2 and 20 weeks of HFD feeding is characterized in Figure 1 and Table 2.

**Table 2:** Body weight, liver weight, and fasting plasma parameters in C57Bl/6 mice fed AIN-93G and HFD for 2 and 20 weeks.

	2 weeks		20 weeks	
	AIN-93G	HFD	AIN-93G	HFD
Body weight [g]	29.35 ± 0.27	32.67 ± 3.99^ns^	32.19 ± 3.78	51.45 ± 1.64^∗^
Liver weight [% b.w.]	5.48 ± 0.77	4.91 ± 0.56^ns^	3.96 ± 0.36	5.47 ± 1.30^∗^
ALT [U/L]	28.05 ± 4.45	40.03 ± 13.27^ns^	67.55 ± 37.76	228.98 ± 86.15^∗^
AST [U/L]	81.66 ± 2.20	64.61 ± 5.61^∗^	125.91 ± 14.43	212.37 ± 59.71^∗^
CHOL [mmol/L]	3.57 ± 0.23	3.83 ± 0.60^ns^	2.93 ± 0.83	4.92 ± 1.16^∗^
HDL [mmol/L]	2.00 ± 0.15	2.10 ± 0.40^ns^	1.61 ± 0.42	1.93 ± 0.32^ns^
LDL [mmol/L]	0.44 ± 0.13	0.43 ± 0.02^ns^	0.20 ± 0.09	0.57 ± 0.19^∗^
TG [mmol/L]	0.99 ± 0.38	0.63 ± 0.15^ns^	0.46 ± 0.20	0.40 ± 0.13^ns^

*Values are means ± SD (n = 3–8 animals per group). ^∗^P < 0.05, ^∗∗^P < 0.01, ^∗∗∗^P < 0.001 significantly different from HFD feeding time-matched AIN-93G group values. AIN-93G, AIN-93G diet fed group; HFD, high fat diet fed group; ALT, alanine aminotransferase; AST, aspartate transaminase; CHOL, total cholesterol; HDL, high density cholesterol; LDL, low density cholesterol; TGs, triglycerides.*

Early phase of NAFLD in HFD-fed mice was not associated with changes in body or liver weight (Table 2). No differences in lipid profile (CHOL, LDL, HDL, TG) or ALT were observed (Table 2). However, the GTT (Figure 1A) indicated development of insulin resistance, despite a lack of difference in plasma fasting glucose (Figure 1A). Histological examination of liver samples showed mild, microvesicular hepatic steatosis without immune cell infiltration or fibrosis (Figure 1B–D).

In the late phase of NAFLD, HFD-fed mice gained significantly more weight and had increased liver weight (Table 2). Plasma concentrations of CHOL, LDL and liver enzymes (AST, ALT), but not HDL and TG, were also significantly elevated (Table 2). Fasting plasma glucose concentration and glucose plasma response during the GTT were significantly increased, indicating severe hyperglycemia and insulin resistance in mice fed HFD for 20 weeks (Figure 1A). Histological analysis displayed prominent macrovesicular liver steatosis without inflammation or fibrosis (Figure 1B–D).

### Pro-inflammatory Phenotype in LSECs in Early and Late Phases of NAFLD

**FIGURE 2:**
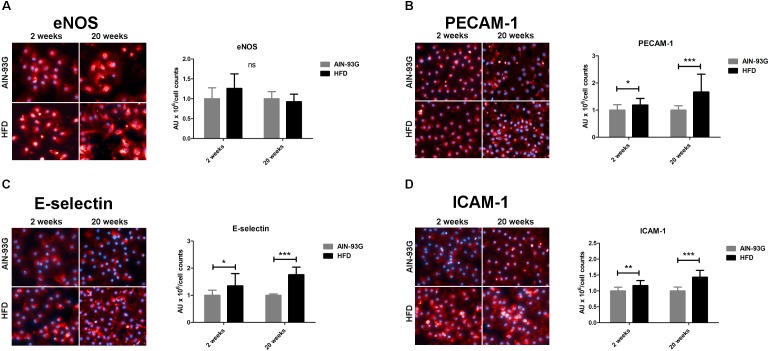
Represenatative immunofluorescence staining and quantitative analysis of immunofluorescence intensity for eNOS **(A)**, PECAM-1 **(B)**, E-selectin **(C)**, and ICAM-1 **(D)** on primary LSECs isolated from C57Bl/6 mice fed AIN-93G and HFD for 2 and 20 weeks. Values are means ± SD. ^∗^*P* < 0.05, ^∗∗^*P* < 0.01, ^∗∗∗^*P* < 0.001 significantly different from HFD feeding time-matched AIN-93G group values. AIN-93G, AIN-93G diet fed group; HFD, high fat diet fed group.

**FIGURE 3:**
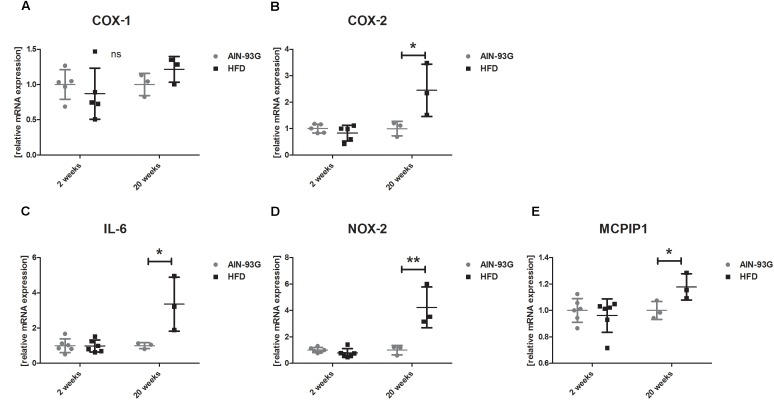
mRNA gene expression of COX-1 **(A)** and COX-2 **(B)**, IL-6 **(C)**, NOX-2 **(D)**, and MCPIP1 **(E)** in primary LSEC isolated from C57Bl/6 mice fed AIN-93G and HFD for 2 and 20 weeks. Values are means ± SD. ^∗^*P* < 0.05, ^∗∗^*P* < 0.01, ^∗∗∗^*P* < 0.001 significantly different from HFD feeding time-matched AIN-93G group values. AIN-93G, AIN-93G diet fed group; HFD, high fat diet fed group; COX-1, cyclooxygenase-1; COX-2, cyclooxygenase-2; IL-6, interleukin 6; NOX-2, NADPH oxidase-2; MCPIP1, monocyte chemotactic protein-1-induced protein-1.

**FIGURE 4:**
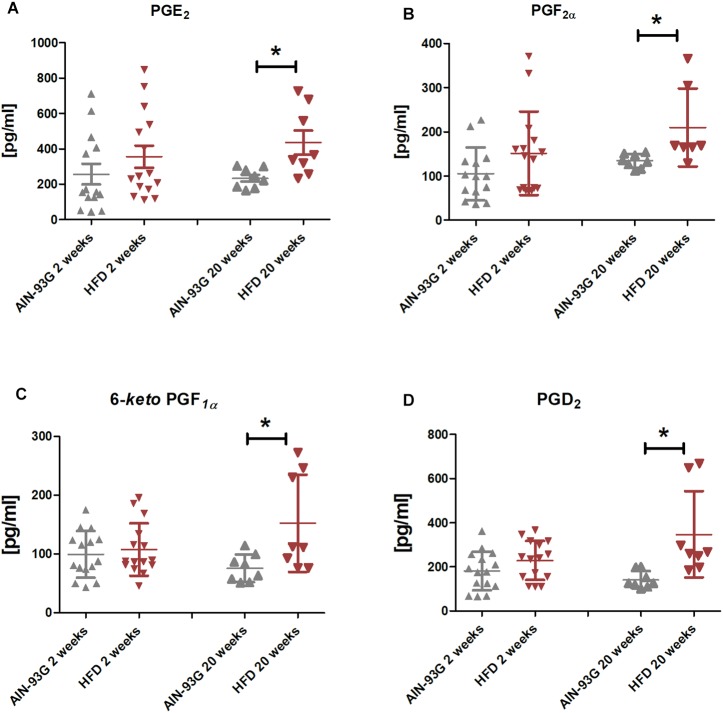
PGE_2_ **(A)**, PGF_2α_ **(B)**, 6-keto-PGF_1α_ **(C)**, and PGD_2_ **(D)** production by primary LSEC isolated from C57Bl/6 mice fed AIN-93G and HFD for 2 and 20 weeks. Values are means ± SD. ^∗^*P* < 0.05, ^∗∗^*P* < 0.01, ^∗∗∗^*P* < 0.001 significantly different from HFD feeding time-matched AIN-93G group values. AIN-93G, AIN-93G diet fed group; HFD, high fat diet fed group.

In the early stage of NAFLD, LSECs displayed increased PECAM-1, E-selectin and ICAM-1 expression (Figure 2B–D) without changes in mRNA expression of pro-inflammatory genes (Figure 3A–D) or prostanoid production (Figure 4A–D). In turn, in the late phase of NAFLD, there was a prominent induction of LSECs’ pro-inflammatory phenotype characterized by increased PECAM-1, E-selectin, and ICAM-1 expression (Figure 2B–D) and significantly increased mRNA expression of COX-2, IL-6, NOX-2 (Figure 3B–D) and production of pro-inflammatory prostanoids such as PGE_2_ and PGF_2a_ (Figure 4A,B). Interestingly, LSECs’ pro-inflammatory activation was compensated by MCPIP1 mRNA overexpression (Figure 3E) and increased secretion of anti-inflammatory PGI_2_ (measured as 6-keto-PGF_1α_) and PGD_2_ (Figure 4C,D) in the late, but not in the early stage of NAFLD. During early and late phase NAFLD progression, no significant changes in nitrite and TXB_2_ production examined *ex vivo* in isolated perfused mice liver were observed (data not shown). In early and late phases of NAFLD, LSEC expression of eNOS (Figure 2A) or COX-1 (Figure 3A) did not change.

### LSEC Fenestrae in Early and Late Phases of NAFLD

**FIGURE 5:**
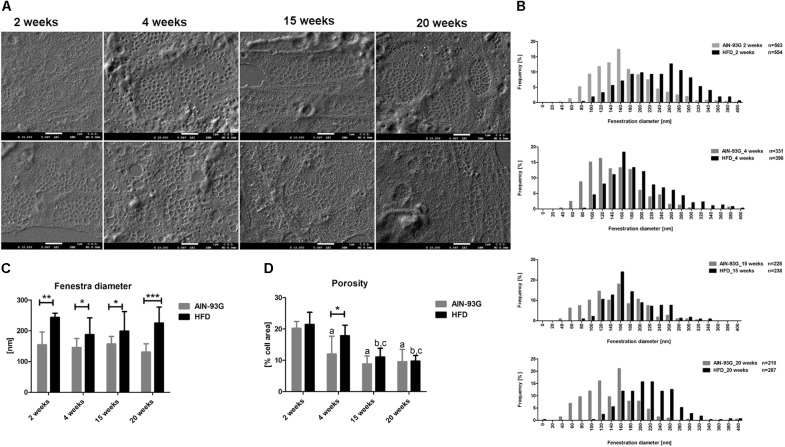
Morphological changes in primary LSECs isolated from C57Bl/6 mice during NAFLD progression evaluated by SEM technique. Representative microphotographs of LSECs sive plates with fenestrae **(A)**, histograms of LSECs fenestrae diameters **(B)**, LSECs mean fenestrae diameter **(C)**, and porosity **(D)**. Values are means ± SD. ^∗^*P* < 0.05, ^∗∗^*P* < 0.01, ^∗∗∗^*P* < 0.001 significantly different from HFD feeding time-matched AIN-93G group values. (a) *P* < 0.05 AIN-93G 2 weeks vs. AIN-93G 4, 15, and 20 weeks, (b) *P* < 0.05 HFD 2 weeks vs. HFD 15 and 20 weeks, (c) *P* < 0.05 HFD 4 weeks vs. HFD 15 and 20 weeks. AIN-93G, AIN-93G diet fed group; HFD, high fat diet fed group.

In order to determine changes in LSECs morphology, SEM and AFM techniques were used. SEM analysis revealed that LSEC fenestrae were preserved at all stages of NAFLD progression (Figure 5A). In detail, analysis of the frequency distribution of LSEC fenestra diameters showed larger diameter of fenestrae in HFD as compared to the AIN-93G group at each stage of NAFLD progression (Figure 5B). Mean diameter of LSEC fenestrae was also larger in the HFD group at each experimental time point (Figure 5C). However, overall LSEC porosity, apart from 4 weeks, did not differ between experimental groups during NAFLD progression (Figure 5D). Interestingly, despite the fact that LSECs porosity was lower in the AIN-93G than the HFD group at 4 weeks (Figure 5D), in general LSEC fenestrae diameter stayed preserved in both groups and did not change with disease progression or animal aging (Figure 5D).

**FIGURE 6:**
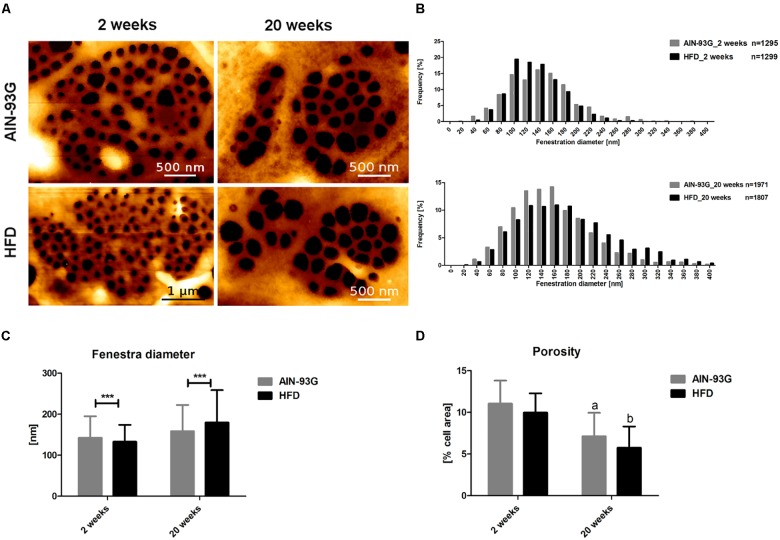
Morphological changes in primary LSECs isolated from C57Bl/6 mice during NAFLD progression evaluated by AFM technique. Representative microphotographs of LSECs sive plates with fenestrae **(A)**, histograms of LSECs fenestrae diameters **(B)**, LSECs mean fenestrae diameter **(C)**, and porosity **(D)**. Values are means ± SD. ^∗^*P* < 0.05, ^∗∗^*P* < 0.01, ^∗∗∗^*P* < 0.001 significantly different from HFD feeding time-matched AIN-93G group values. (a) *P* < 0.05 AIN-93G 2 weeks vs. AIN-93G 20 weeks, (b) *P* < 0.05 HFD 2 weeks vs. HFD 20 weeks. AIN-93G, AIN-93G diet fed group; HFD, high fat diet fed group.

Morphology measurements with AFM were in line with results obtained by SEM because they confirmed that LSEC fenestrae were preserved even at the late stage of NAFLD in HFD mice (Figure 6A). Accordingly, differences in LSEC porosity between experimental groups at early and late stage of NAFLD were not found either in SEM or AFM data (Figure 5D, 6D). Interestingly, in contrast to SEM, AFM measurements showed practically the same mean fenestrae diameters in HFD and AIN-93G groups at the early phase of NAFLD. Furthermore, both techniques identified a significantly increased number of LSECs with bigger fenestrae (up to 400 nm in diameter) in the HFD group for the late stage of disease (Figure 6B,C). Importantly, because the AFM technique allows us for observation and measurements of dynamic fenestrae rearrangements (Braet and Wisse, 2017; Zapotoczny et al., 2018), we stress that analyzed fenestrae with diameters up to 400 nm are fully functional and therefore taken for analysis. Moreover, similarly to SEM results, the AFM method showed diminished LSEC porosity related with aging (Figure 6D).

### LSEC Bioenergetics in the Early and Late Stages of NAFLD

**FIGURE 7:**
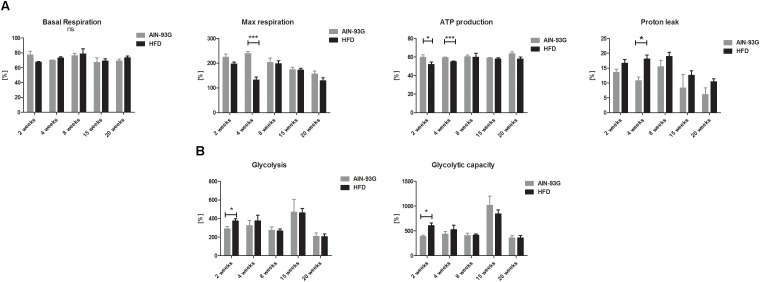
Basal OCR, ATP production, maximal respiration, and mitochondrial proton leak **(A)** and basal ECAR, glycolysis, glycolytic capacity **(B)** in primary LSEC isolated from C57Bl/6 mice fed AIN-93G and HFD for 2 and 20 weeks. Values are means ± SD. ^∗^*P* < 0.05, ^∗∗^*P* < 0.01, ^∗∗∗^*P* < 0.001 significantly different from HFD feeding time-matched AIN-93G group values. AIN-93G, AIN-93G diet fed group; HFD, high fat diet fed group.

Liver sinusoidal endothelial cell bioenergetics were largely preserved at early as well as in late stages of NAFLD (Figure 7A,B). Visible in early NAFLD mild impairment of mitochondrial phosphorylation (decreased ATP production, impaired maximal mitochondrial respiration and increased proton leak) (Figure 7A) was compensated by concomitant increased LSEC basal glycolysis and maximal glycolysis capacity (Figure 7B). Proton leak tended to increase in the NAFLD group, but this difference reached significance only at the time point of 4 weeks of HFD.

## Discussion

In the present study, we demonstrated that in HFD-induced NAFLD in mice, featured by prominent insulin resistance, liver steatosis and obesity, LSECs displayed a pro-inflammatory phenotype that was however associated with fully preserved LSEC fenestrae and bioenergetics. These findings shed novel light on the relationship between LSEC pro-inflammatory response, fenestrae status and bioenergetics in NAFLD development in mice.

Under physiological conditions, LSECs are characterized by the presence of fenestrae and lack of a basement membrane. However, liver inflammation and fibrosis are associated with loss of fenestrae and formation of a continuous basement membrane, the latter due to deposition of extracellular matrix in the space of Disse (Maslak et al., 2015a). The phenomenon of LSEC defenestration is known to contribute to various liver pathologies (Mori et al., 1993; Steffan et al., 1995; Yamamoto et al., 1996; Wang et al., 2005) and was also suggested to play a pivotal role in NAFLD progression known to involve LSEC dysfunction (Miyao et al., 2015). However, it is not clear whether LSEC inflammatory response is linked with LSECs’ defenestration. Therefore, in the current study we re-evaluated this relationship and demonstrated that in the early phase of NAFLD (after 2 weeks of HFD), LSECs displayed a mild pro-inflammatory phenotype, while pronounced inflammatory activation was noticed at the late stage of NAFLD (20 weeks of HFD). Importantly, LSEC inflammation was not related to their defenestration or impaired bioenergetics.

Previously, a number of reports have demonstrated that intralobular inflammation, hepatic fibrosis, and cirrhosis are characterized by the presence of defenestrated LSECs (Dobbs et al., 1994; Straub et al., 2007). Furthermore, liver recovery in thioacetamide (TAA)-induced and in CDAA-induced murine models of NASH were related to the reversal of LSEC defenestration (Xie et al., 2012; Miyao et al., 2015). On the other hand, the relation of LSEC inflammation in the process of defenestration and its contribution in the pathophysiology of NAFLD was not elucidated previously. Miyao et al. (2015), using CDAA, and HFD models of NASH/NAFLD, suggested that LSEC defenestration was observed in both of them. In a CDAA model, LSEC defenestration preceded the appearance of inflammation and fibrosis, although activation of Kupffer cells and HSCs at the transcriptional level was already observed at the stage of LSEC defenestration. On the other hand, in an HFD model, LSEC capillarization was absent until occurrence of intralobular inflammatory cell infiltration and fibrosis present after 22 weeks of experimental feeding in the work of Miyao et al. (2015). In our hands, liver inflammation and fibrosis were virtually absent in the HFD model, in contrast to the work of Miyao et al. (2015). However, a significant LSEC inflammatory response was present, but was not associated with LSEC defenestration. Accordingly, our results extend the previous reports and suggest that LSEC dysfunction and inflammation, as long as HSC-induced fibrosis and LSEC basement membrane formation is maintained, are not necessarily linked to defenestration. Moreover, our data point to the fact, that liver steatosis without infiltrating immune cells, HSC activation and fibrosis was related with increased fenestrae diameter, rather than with defenestration. These findings suggest that LSEC inflammation and defenestration are not necessarily linked to each other; furthermore, LSEC inflammation might be even linked with compensatory increase in LSEC fenestrae diameter.

In our work, we used two complementary methods to analyze fenestrae in primary LSECs isolated from mice fed HFD as well as control mice. We took advantage of SEM, as a standard method used by many authors to study fenestrae morphology (Braet et al., 1996a; Simon-Santamaria et al., 2010; Xie et al., 2012) and the unique method of AFM imaging that has been advanced for LSEC morphology probing (Braet et al., 1996c; Zapotoczny et al., 2017b,c).

Even though, the accuracy of the fenestrae measurement depends on the technique of biological material preparation, that is distinct for SEM and AFM, quite surprisingly, for control mice mean fenestrae diameter of LSEC fenestra was quite similar for both measurements (2 vs. 20 weeks, 155 ± 42 vs. 131 ± 27 nm for SEM and 142 ± 58 vs. 158 ± 64 nm for AFM, respectively) suggesting that LSECs’ absolute diameter is relatively preserved for control LSECs analyzed under experimental conditions of SEM and AFM measurements. In contrast, porosity values were quite different for SEM vs. AFM (2 vs. 20 weeks, 20 ± 2 vs. 10 ± 4 nm for SEM and 11 ± 3 vs. 7 ± 3 nm for AFM). On the other hand, only SEM data showed increase in fenestrae diameter resulted from HFD feeding at every stage of NAFLD, while the AFM technique showed such an effect only for the late phase of NAFLD. Both techniques identified also that LSEC porosity did not change with the progression of NAFLD, but decreased with age.

Altogether, our results clearly demonstrate that in an HFD-model of NAFLD, LSEC fenestrae are preserved and have increased diameter in HFD in comparison to AIN-93G groups. These results might indicate that excessive fat influx to the liver results in increased LSEC fenestrae diameter increasing the passage of lipids and other molecules to the main place of its metabolism, to hepatocytes possibly protecting against lipotoxicity of other cells in the liver. A similar suggestion was made by Fraser et al. (1980). Authors suggested a beneficial effect of increased porosity of LSECs sieve in the development of acute fatty liver after alcohol consumption. Accordingly, regulation of LSEC fenestrae diameter may play an important adaptive role in lipid transport and metabolism in NAFLD development.

The structure of LSEC fenestrae is maintained by the presence of a sieve plate-associated cytoskeleton and FACRs, connected to a cytoskeletal framework of microfilaments and microtubules (Braet et al., 1995, 1996b) and cytoskeleton reorganization (Steffan et al., 1987); an energy-demanding process, which is involved in fenestrae dynamics (Braet et al., 1996a, 2003; Zapotoczny et al., 2017c). In fact, it was shown that the effect of antimycin A-derived defenestration may be related with diminished ATP production due to inhibition of complex III of the mitochondrial respiratory chain (Braet et al., 2003). In our study, LSEC bioenergetics was largely preserved at early as well as in late stages of NAFLD, suggesting that fully maintained LSEC bioenergetics allowed to provide sufficient supplies for energetic demand of LSECs to preserve fenestrae and modulate their size as needed.

Altogether, our data indicate that even at late stage of HFD-induced NAFLD progression, LSECs’ pro-inflammatory phenotype is related with preserved LSEC bioenergetics and maintained functional fenestrae. As a result, quite surprisingly, we rather observed compensation, than compromise of LSEC function.

In the current study, we showed parallel activation of pro-inflammatory (PGE_2_, PGF_2α_) and anti-inflammatory (PGD_2_, PGI_2_) prostanoids production in LSECs, suggesting a possible offsetting role of LSEC-derived PGI_2_ and PGD_2_ in the development of NAFLD. Similarly, observed activation of MCPIP1 in LSECs, a negative regulator of inflammatory processes in liver (Jura et al., 2012; Lin et al., 2014), most likely was related with induction of inflammation in LSECs, since MCPIP1 undergoes rapid and potent transcription induction upon stimulation with pro-inflammatory molecules, such as MCP-1, IL-1β, TNFα, and others (Skalniak et al., 2009). These findings are in line with the notion that the adaptive phase of LSEC inflammation is usually balanced by anti-inflammatory mechanisms as shown in other models (Gilroy et al., 1999; Praticò, 2008). The hepatoprotective role of PGD_2_ and PGI_2_ was previously demonstrated showing prominent inhibition of HSC activation in ConA-induced hepatitis (Ohta et al., 2005; Fujita et al., 2016) and reducing fructose-induced hepatocellular steatosis (Zhang et al., 2017). Accordingly, these adaptive changes might attenuate LSEC inflammation, modulate function of other type of liver cells and inhibit the development of NAFLD.

Regarding bioenergetics, although HFD transiently impaired LSEC mitochondrial function (decrease in ATP synthesis and diminished maximal mitochondrial respiration) as soon as after 2 weeks of HFD feeding; this effect was related to a compensatory increase in glycolysis and maximal glycolytic capacity in LSECs. This result indicated prominent energetic flexibility of LSECs in order to maintain cells’ bioenergetic balance. Moreover, quite surprisingly, LSEC bioenergetics was fully preserved at the late stage of NAFLD, indicating LSECs’ ability to adapt to ongoing pathological conditions as demonstrated also for kidney mitochondria in a similar model of HFD (Ruggiero et al., 2011). In our work, we also provide evidence that LSEC mitochondria tend to be uncoupled. Previously, it was shown that mitochondria isolated from HFD-fed mice liver exhibited increased ROS production (Matsuzawa-Nagata et al., 2008), compatible with the notion that elevated influx of fatty acids alters bioenergetic functions of liver mitochondria and results in mitochondrial-derived oxidative stress. In the current study, we analyzed bioenergetic response of LSECs to HFD in mice for the first time, and demonstrated that HFD seem to increase LSECs’ mitochondrial proton leak initially, that was later normalized, suggesting transient mitochondrial uncoupling protein-2 (UCP2) overexpression (Evans et al., 2008) or LSEC adaptation to increased influx of fatty acids. However further studies are needed to evaluate role of LSEC mitochondria in lipids metabolism in NAFLD.

In summary, we have demonstrated that in early and in late phase of NAFLD, LSEC fenestrae are functionally preserved despite metabolic and pro-inflammatory burden linked to HFD and NAFLD progression. Furthermore, we identified a number of adaptive mechanisms such as increased production of anti-inflammatory PGI_2_, PGD_2_, overexpression of MCPIP1 and bioenergetic adaptation. These results indicate that LSEC fenestrae preservation in NALFD is associated with biochemical as well as metabolic adaptive mechanisms that may all play an important role mitigating NAFLD progression.

## Author Contributions

EK and SC: study conception and design. EK, PK, IC-C, KS, BZ, AK, AS, JK, and LM: acquisition of data. EK, PK, IC-C, KS, BZ, LM, EC, and SC: analysis and interpretation of data. EK: drafting of the manuscript. SC and MS: critical revision.

## Conflict of Interest Statement

The authors declare that the research was conducted in the absence of any commercial or financial relationships that could be construed as a potential conflict of interest.

## Abbreviations

2DG: 2-deoxy-D-glucose
AFM: atomic force microscopy
Akt: protein kinase B
ALT: alanine aminotransferase
AST: aspartate aminotransferase
AUC: area under the curve
CDAA: choline-deficient, L-amino acid-defined diet
CHOL: total cholesterol
COX-2: cyclooxygenase-2
ECAR: extracellular acidification rate
eNOS: endothelial nitric oxide synthase
FACRs: fenestrae-associated cytoskeleton rings
GTT: glucose tolerance test
HDL: high-density lipoprotein cholesterol
HFD: high fat diet
HSCs: hepatic stellate cells
ICAM-1: intercellular adhesion molecule 1
IL-6: interleukin 6
LDL: low-density lipoprotein cholesterol
LSECs: liver sinusoidal endothelial cells
MCPIP1: monocyte chemotactic protein-1-induced protein-1
NAFLD: non-alcoholic fatty liver disease
NASH: non-alcoholic steatohepatitis
NO: nitric oxide
NOX-2: NADPH oxidase 2
OCR: oxygen consumption rate
ORO: Oil Red O
PECAM-1: platelet endothelial cell adhesion molecule-1
PGD_2_: prostaglandin D_2_
PGE_2_: prostaglandin E_2_
PGF_2α_: prostaglandin F_2α_
PGI_2_: prostacyclin
PSR: Picro Sirius Red
SEM: scanning electron microscopy
TGs: triglycerides
TXA_2_: thromboxane A_2_
TXB_2_: thromboxane B_2_.
